# A roadmap for designing narrow-spectrum antibiotics targeting bacterial pathogens

**DOI:** 10.15698/mic2022.07.780

**Published:** 2022-07-04

**Authors:** Xinyun Cao, Robert Landick, Elizabeth A. Campbell

**Affiliations:** 1 Department of Biochemistry, University of Wisconsin-Madison; Madison, United States.; 2 Department of Bacteriology, University of Wisconsin-Madison; Madison, United States.; 3 Laboratory of Molecular Biophysics, The Rockefeller University; New York, United States.

**Keywords:** narrow-spectrum antibiotics, RNA polymerase, transcription, Clostridioides difficile, fidaxomicin

## Abstract

*Clostridioides difficile* (*Cdiff*) infection (CDI) continues to be the leading threat of nosocomial deaths worldwide and a major burden on health-care systems. Broad-spectrum antibiotics eradicate the normal gut microbiome, killing protective commensal bacteria and increasing CDI recurrence. In contrast, Fidaxomicin (Fdx) is a narrow-spectrum antibiotic that inhibits *Cdiff* growth without affecting crucial gut microbes. However, the basis of the narrow-spectrum activity of Fdx on its target, RNA polymerase (RNAP), in *Cdiff* has been enigmatic. Recently, Cao *et al.* (Nature, doi: 10.1038/s41586-022-04545-z) combined transgenic RNAP design and synthesis with cryo-electron microscopy (cryo-EM) to identify a key determinant of Fdx inhibition of *Cdiff* RNAP. This finding was further corroborated by biochemical, bioinformatics, and genetic analysis. This microreview describes implications of this work for lineage-specific antibiotic design and new directions toward understanding transcription and regulation in *Cdiff* and other bacterial pathogens.

## Fdx INHIBITS RNAP TRANSCRIPTION ACTIVITY

**Figure 1:**
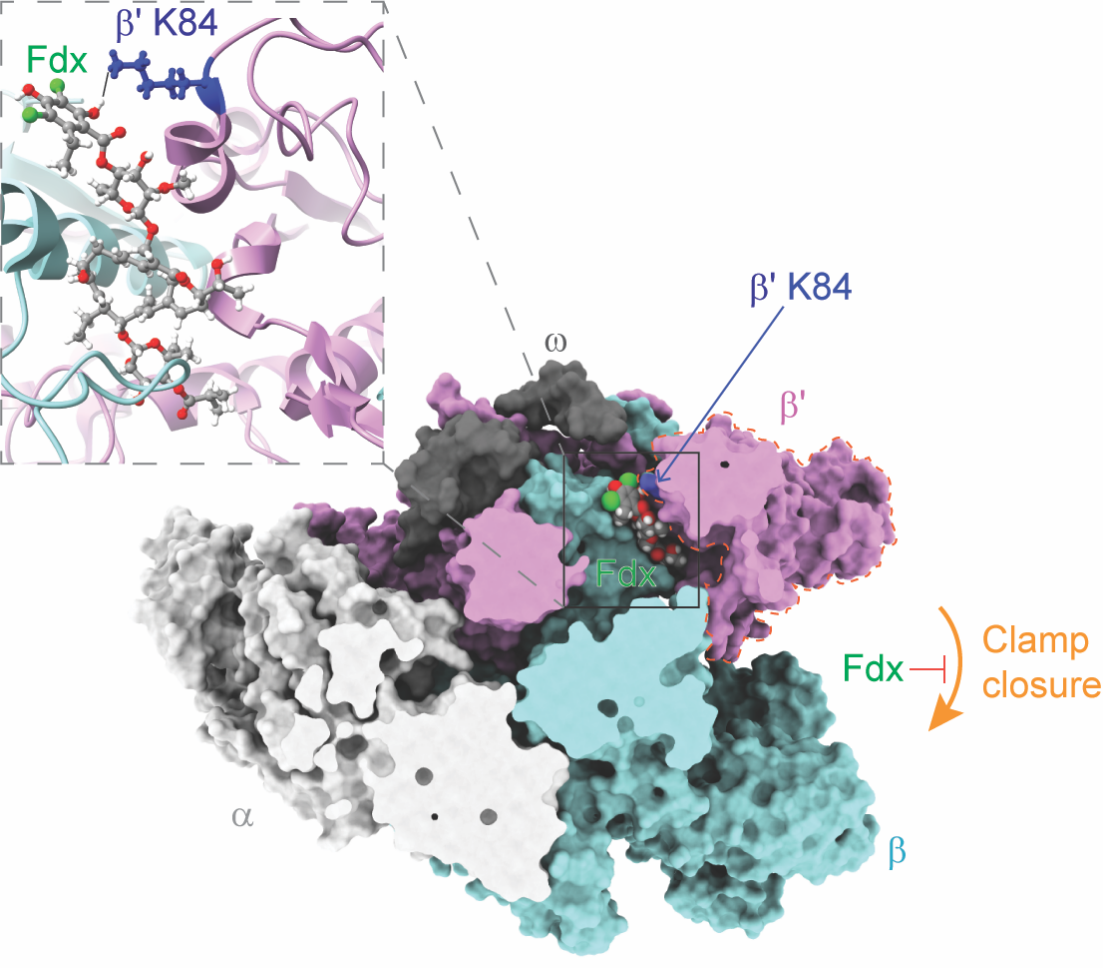
FIGURE 1. Fdx is a narrow-spectrum antibiotic inhibiting *Cdiff* RNA polymerase. Fdx (shown in ball sphere) fits in the narrow gap between β and β' subunit of *Cdiff* RNAP and jams the RNAP clamp (dash line region) in an open state. The structure is sliced at the level of the Fdx-binding pocket. Note that the Sigma A factor was removed from the structure (PDB: 7L7B) for visualization purposes. Inset, magnification of Fdx sensitizer residue β'K84 interacting with the hydrophilic group of Fdx. Fdx and β'K84 residue are shown in ball-and-stick representations.

RNAP transcribes genetic information from DNA to RNA, and has been a proven drug target for decades. RNAP is large protein machinery with mobile pincers that must move to load DNA for transcription. Based on the cryo-EM structure of *Cdiff* RNAP in complex with Fdx, Cao, *et al.*, found that Fdx squeezed into the hinge between the two pincers formed by the β and β' subunits of *Cdiff* RNAP, jamming one of the pincers, known as the RNAP clamp, open. This jamming prevents the clamp from closing and prevents RNAP from grabbing onto the DNA to start the transcription process (**Figure 1**). The general mechanism of Fdx's inhibition of *Cdiff* RNAP is similar to that previously observed in *Mycobacterium tuberculosis* (*Mtb*) RNAP.

## BACTERIA'S RELEVANT BIOLOGICAL NICHES AND MICROBIAL COMPETITORS MUST BE CONSIDERED FOR NARROW-SPECTRUM ANTIBIOTICS DISCOVERY

*Cdiff* can colonize the gastrointestinal tract and interacts with its natural inhabitants (*i.e.,* commensal gut bacteria). A healthy human gut microbiome is rich in microbial diversity and abundance. By competition for resources in this niche, the commensals keep the population of *Cdiff* in check and therefore tempers *Cdiff* colonization and proliferation. When the gut community is disrupted, for example, by broad-spectrum antibiotics, *Cdiff* colonization can progress and cause infection. To understand the action of Fdx in the context of the gut microbiome, *Cao et al.* generated a “roadmap” using the structure of *Cdiff* RNAP in complex with Fdx. In this “roadmap,” every Fdx-binding residue is a “destination.” After analyzing the conservation of each “destination” in RNAP of common gut species by comparative sequence alignment, *Cao et al.* pinpointed one amino-acid residue (β'K84 in *Cdiff* RNAP) that appears to play a crucial role in determining Fdx sensitivity in the gut microbiome. Thus, this residue is called the Fdx sensitizer. In the Fdx-sensitive phyla Firmicutes and Actinobacteria, the sensitizer residue is always positively charged (lysine or arginine) and forms a tight interaction with a negatively charged phenolic group of Fdx (**Figure 1**). However, in Bacteroidetes, an abundant species in the gut microbiota that protects against *Cdiff* colonization, the sensitizer position is negatively charged glutamic acid, leading to repulsion of Fdx. In Proteobacteria, the sensitizer position is a neutral residue (*e.g.,* glutamine or serine), resulting in less-tight binding to Fdx. Both Bacteroidetes and Proteobacteria are Fdx-resistant. Cao *et al.* then biochemically and biologically validated the Fdx-sensitizer residue as a crucial contributor to the narrow spectrum activity of Fdx. They proposed that the Fdx-binding residues in the “roadmap” can also be used to predict Fdx potency in other bacteria.

## THE STRUCTURAL AND MECHANISTIC STUDIES OF Fdx CONTACTING *Cdiff* RNAP INFORM EFFORTS TO DESIGN LINEAGE-SPECIFIC ANTIBIOTICS RATIONALLY

The rapid emergence and prevalence of new antibiotic-resistant isolates for CDI treatment under current therapies (metronidazole, vancomycin, *etc.*) have caused great alarm in the medical community. Therefore, the need to develop new antibiotics that kill *Cdiff* but do not cause collateral damage to the gut microbiome is compelling. The “roadmap” of Fdx-contacting residues in *Cdiff* RNAP can guide novel drug discovery in two approaches: *in silico–*guided and experimental. In the *in silico*–guided path, the Fdx-binding region in *Cdiff* RNAP can serve as an identification target for docking-based virtual screening of available and novel compounds. Furthermore, the screening data could provide valuable information for training ligand-based models (*i.e.,* machine learning). During this process, parameters can be set to select compounds interacting with the sensitizer residue for Fdx-binding specificity among bacterial lineages. Note that a candidate compound may have a completely different inhibitory mechanism even when sharing the same binding pocket with Fdx because RNAP transcription initiation is highly dynamic. Hence, further biochemical and structural studies would be required to characterize their mechanisms. In the experimental path, Fdx can be rationally modified toward inhibiting selected pathogens by chemical synthesis. For example, to improve Fdx potency for *Cdiff* (*i.e.,* increase drug–RNAP binding affinity), one might be able to substitute the phenolic group of Fdx, which interacts with the sensitizer residue (**Figure 1**), with a stronger acid. To inhibit gram-negative pathogens while not affecting gram-positive bacteria, one could replace the phenolic group with a basic group. *In vitro*, Fdx is less permeable to gram-negative bacteria because of the outer membrane barrier. Nevertheless, adopting the Fdx modification strategy in combination with an outer membrane weakener is promising for treating gram-negative pathogens.

## THE ROLE OF TRANSCRIPTION FACTORS (TF) IN Fdx SENSITIVITY IN BACTERIA

The transcription process of RNAP is highly dynamic and is regulated by numerous TFs. Many TFs participate in unique regulatory mechanisms in pathogenic bacteria and may contribute to antibiotic sensitivity. For example, *Mtb* RbpA is an essential TF that stabilizes the RNAP–promoter open complex (RPo) and directly contacts Fdx. Previous biochemical studies established that RbpA sensitized *Mtb* to Fdx by one order of magnitude *in vitro*. Meanwhile, mutation of another essential TF in *Mtb*, CarD, increases Fdx sensitivity *in vivo,* possibly by causing a defect in stabilizing RPo. *Cdiff* lacks RbpA but has CarD. It will be important to understand how CarD and other TFs involved in transcription initiation alter fidaxomicin sensitivity in *Cdiff*.

## THE SYNTHETIC SYSTEM OPENS A DOOR FOR ELUCIDATION OF THE MOLECULAR MECHANISMS OF TRANSCRIPTION AND REGULATION IN PATHOGENS

Many bacterial pathogens like *Cdiff* have different RNAP transcriptional features and regulators compared to model organisms *E. coli* and *B. subtilis*. These unique features can be exploited to design narrow-spectrum antibiotics for combating infectious diseases. However, it is difficult to purify RNAP directly from many pathogens because their purification requires bacterial growth at a large scale with specific biosafety measures to yield enough enzyme. Meanwhile, the sparse genetic tools for pathogen manipulation make mechanistic studies difficult. Therefore, a synthetic expression system was devised by Cao *et al.* to make recombinant *Cdiff* RNAP in *E. coli* by combining the genes for all four subunits on a single plasmid with appropriate tags that enabled rapid purification of the recombinant enzyme. The *E. coli* expression system provides high yields of *Cdiff* RNAP suitable for biochemical and structural studies and enables rapid mutagenesis, which would be impossible for lethal substitutions in the much less genetically tractable native *Cdiff*. Furthermore, this expression system defines a route for similar studies with other pathogenic bacteria.

